# Beneficial outcomes of medication review in patients with hip fractures: a systematic review

**DOI:** 10.1007/s41999-025-01252-6

**Published:** 2025-06-19

**Authors:** Dina Aprillia Ariestine, Taro Kojima, Fakhriza Hidayati Siregar, Alvin Ivander

**Affiliations:** 1 Division of Geriatric Medicine, Department of Internal Medicine, Faculty of Medicine, Universitas Sumatera Utara, Medan, Indonesia; 2 Department of Geriatric Medicine, International University of Health and Welfare, Narita, Japan; 3 Faculty of Medicine, Universitas Sumatera Utara, Medan, Indonesia

**Keywords:** Medication review, Polypharmacy, Hip fractures, Older adults

## Abstract

**Purpose:**

This study aims to analyze medication review efficacy toward clinical outcomes in older adults with hip fractures.

**Methods:**

A systematic search was conducted using the modified keywords “medication review,” “polypharmacy,” and “hip fractures” to find relevant studies. Following the PRISMA guidelines, we reviewed published studies from 2014 to 2024. A total of 21 studies have been identified, six of which reported medication reviews for older adults with hip fractures.

**Results:**

The findings highlight the critical need for a multi-faceted strategy to address the challenges of polypharmacy in older adults with hip fractures. A higher number of medications is associated with some adverse outcomes, such as increased hospitalization rates, longer recovery times, and a greater incidence of pressure ulcers. Although minimizing inappropriate medications may show some positive results, focusing solely on medication management is insufficient in this vulnerable population.

**Conclusion:**

Medication reviews have substantially mitigated medication-related issues, including costs, drug efficacy, and patient discomfort. Nevertheless, clinical outcomes have yet to exhibit significant improvement, which presents an opportunity for further research to explore the potential benefits of medication reviews in enhancing the care of patients with hip fractures. A holistic approach, which consists of medication review, patient education, and tailored rehabilitation programs, may be essential to improve recovery, decrease hospitalization rates, and prevent adverse events.

## Key summary points

**Aim:**

The aim of this study is to evaluate the advantages of medication reviews in older adults with hip fractures and polypharmacy.

**Findings:**

Medication reviews significantly reduced inappropriate prescribing, the incidence of medication-related problems, and post-operation complications, albeit they were not associated with lower mortality or hospitalization rates.

**Message:**

Conducting medication reviews is essential for enhancing medication management and health outcomes in the geriatric population despite the lack of a significant reduction in mortality rates.

## Introduction

The majority of hip fractures in older adults result from falls, with a striking 95% of this population having a history of such incidents. These falls are not isolated incidents but often reflect broader health challenges associated with aging. Such challenges arise from age-related changes in organ function, underlying diseases, and environmental factors [1]. Conditions such as gait and balance disorders, frailty, disability, comorbidities, and the complexities associated with polypharmacy contribute significantly to the risk of falls in this population [2, 3]. Notably, older adults who have experienced a fall in the past year face a considerably higher risk of recurrent falls [4]. The recurrence of falls not only raises the likelihood of fractures but also has devastating consequences, particularly for those suffering from frailty and osteoporosis [5].

A critical concern for older adults who experience falls or hip fractures is the increased likelihood of polypharmacy. Defined as the concurrent use of five or more medications, polypharmacy often emerges as patients transition through different therapeutic settings [4]. To manage pain, prevent complications, and address comorbid conditions, many healthcare providers may prescribe multiple medications [6]. However, this necessary medical intervention can be a double-edged sword. While medications play a vital role in treatment and recovery, they also elevate the risk of falls. Alarmingly, two-thirds of the older adult population are prescribed combinations of medications known to increase this risk, emphasizing the need for careful medication management [7–9].

One effective strategy to mitigate the risks associated with polypharmacy is a structured medication review (MR). This process involves a comprehensive assessment of a patient’s medications to optimize therapeutic outcomes, minimize adverse effects, and ensure individualized prescribing [10, 11]. Regular medication reviews are particularly crucial for older adults with polypharmacy, as they help identify medication-related problems and facilitate appropriate interventions. A structured review should utilize designated tools to assess quality of life, functionality, primary care objectives, and life expectancy [12].

Given the strong interconnection between falls, hip fractures, polypharmacy, and medication safety, comprehensive assessments and targeted interventions, such as medication reviews, are essential for health improvements in this vulnerable population. Recognizing this critical need, the authors conduct a systematic review to explore the benefits of medication review for older adults with hip fractures undergoing polypharmacy.

## Methods

This systematic review aims to investigate the beneficial outcomes of medication reviews to prevent polypharmacy among patients with hip fractures. Our systematic review is registered in PROSPERO CRD42023463636. [x] This study followed the Preferred Reporting Items for Systematic Reviews and Meta-Analyses (PRISMA) 2020 guidelines.

Reference X: Page MJ, McKenzie JE, Bossuyt PM, Boutron I, Hoffmann TC, Mulrow CD, Shamseer L, Tetzlaff JM, Akl EA, Brennan SE, Chou R. The PRISMA 2020 statement: an updated guideline for reporting systematic reviews. bmj. 2021 Mar 29;372.

### Search strategies

The search strategies were developed in collaboration with a librarian, focusing on keywords related to “medication review,” “polypharmacy,” and “hip fractures.” A comprehensive literature search was conducted across several electronic databases, including Oxford, PubMed, SpringerLink, EBSCO, and Google Scholar. Hand-searching and gray literature were excluded to avoid biased studies or those lacking any scientific scrutiny. Keywords were adjusted as needed to comply with the specific requirements of each database, thereby optimizing the literature search process. The inclusion criteria for this review were observational or experimental studies (1), medication review as the intervention (2), and older adults (≥ 65 years old) with hip fractures as the target population. Systematic reviews (1), literature reviews (2), and case report studies (3) were excluded. All article titles and abstracts were screened for potential relevance, and those identified as relevant were further assessed by reviewing their full texts against the established eligibility criteria. Despite the fact that this review has no geographic restrictions, only English-language articles were included. Our review has no publication time limitation as we wish to collect a broad range of high-quality studies even from previous decades. In our review, polypharmacy is defined as the use of five or more different medications scheduled and administered to a single patient during their care.

### Data extraction

After removing duplicate entries, two review authors independently examined all abstracts and full-text articles to determine their relevance based on the established inclusion and exclusion criteria. Data from the eligible publications were then extracted centrally. The information extracted contained the following standardized details from the included articles: the first author’s name, the publication year, the study design, the patient numbers per group, the outcomes measured, the key findings, and the follow-up period. The extracted findings for this study comprised mortality rates and hospitalization occurrences.

### Risk-of-bias assessment

**Fig. 1:**
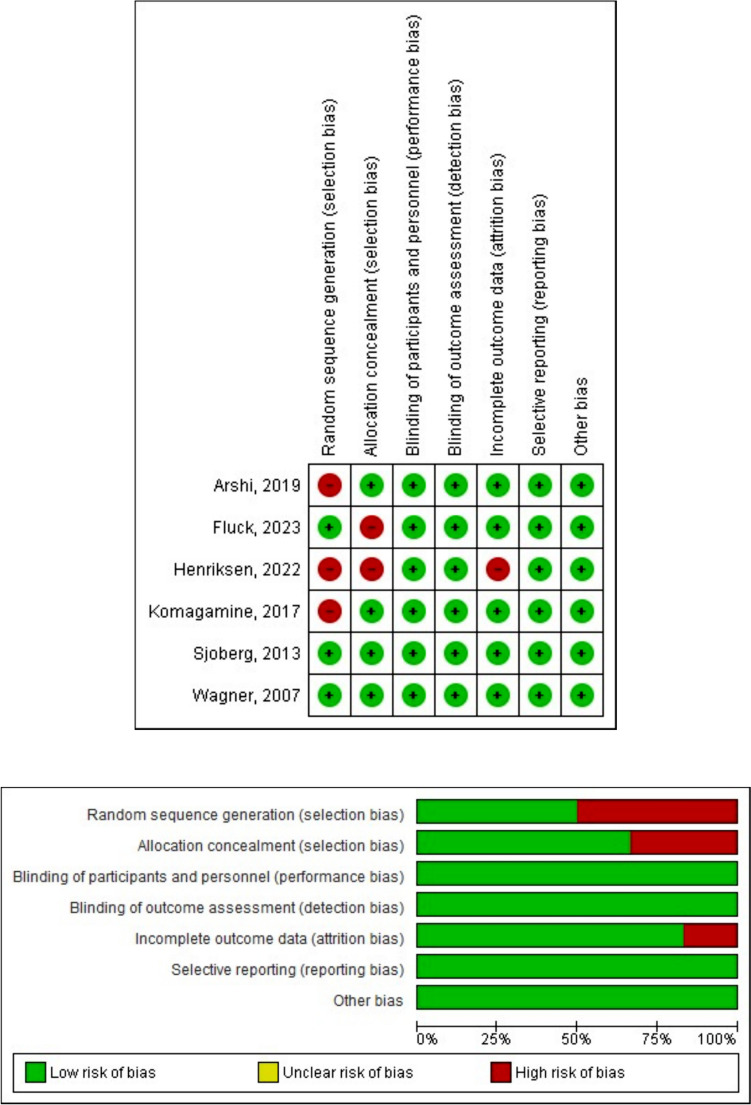
ROBINS-2 (Risk of Bias Interventional Study) assessment of included studies

To align with the study’s objectives, we utilized the ROBINS-2 tool to systematically measure the risk of bias (RoB) and address any concerns regarding the relevance of the selected literature for our research (Fig. 1). Any disagreements that arose during this process were resolved through discussion, and unresolved cases were adjudicated by the most senior reviewers after considering the perspectives of all authors, reviewers, and the manuscript itself. Heterogeneity could not be assessed due to inherent principles. Medication review itself as a practice is done differently according to the respective healthcare system.

## Results

### Search results

**Fig. 2:**
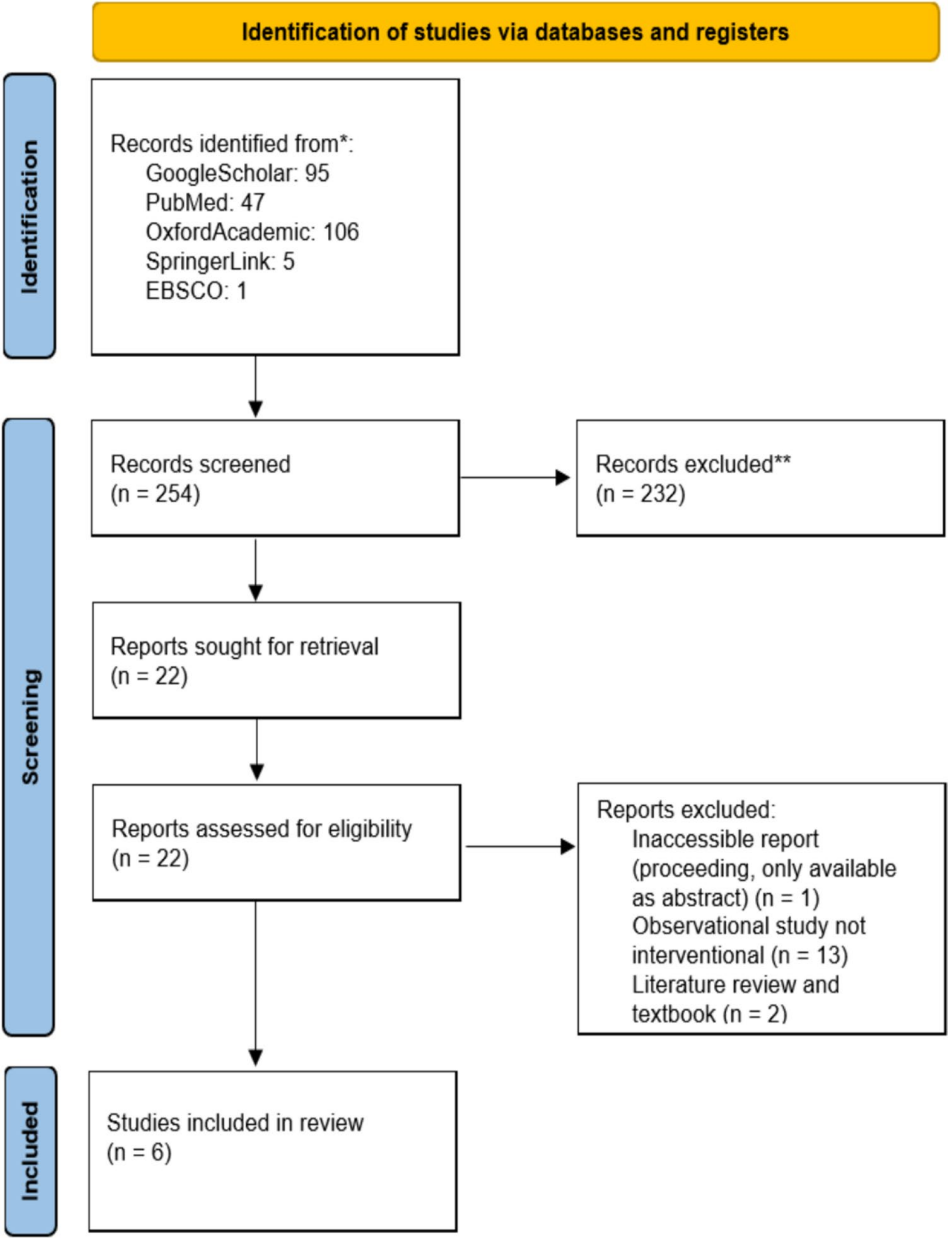
PRISMA flow-chart of study recruitment

We identified 253 articles from four databases and excluded 232 articles due to database duplication and non-relevance. Then, we reviewed the full text of 22 other articles, revealing that one could not be accessed, 13 focused solely on the risk of polypharmacy instead of interventional attempts, and two were textbooks and literature reviews. Our process can be reviewed in Fig. 1. Furthermore, a RoB assessment was also conducted to measure the review evidence, as seen in Fig. 2. The ROBINS-2 assessment result showed that most studies had a high risk of bias. Notably, the Henriksen study did not even report patient outcomes, which would embolden bias; however, to preserve study numbers, this could not be excluded.

### Medication review beneficial outcome

#### Impact of individual clinician-based medication review

We procured six studies with heterogeneous methods and outcomes. Fluck et al. categorized the geriatric hip fracture participants into three levels of medication status based on the number of daily drugs consumption according to the Anticholinergic Burden (ACB) scale. The percentage of patients hospitalized for 1 week or longer varied among the groups: 55.1% for level 1, 76.6% for level 2, and 80.9% for level 3. The percentage of patients admitted to the hospital for 2 weeks or more increased from 19.8% (level 1) to 31.5% (level 2) and 38.1% (level 3), and those unable to move within 1-day post-surgery also rose from 23.2% (level 1) to 36.2% (level 2) and 43.2% (level 3). In addition, the rate of pressure ulcers escalated from 0.4% (level 1) to 1.3% (level 2) and 3.6% (level 3). Consequently, the authors concluded that polypharmacy and/or ACB were associated with adverse impacts in hip fracture patients, including longer hospital stays, comorbidities (such as dementia, stroke, ischemic heart disease, and diabetes), failure to mobilize within 1-day after surgery, and an increased occurrence of pressure ulcers.

Fluck et al. provided additional evidence regarding the effects of polypharmacy, particularly in patients with an ACB, and supported the need to decrease potentially inappropriate prescriptions (PIPs). This study is insightful due to the number of samples included. Fluck et al. were able to analyze 1105 samples in total, which strengthened the validity of polypharmacy’s role in hip fracture consequences.

Komagamine et al.’s study was consistent with the findings of Sjoberg and Wallestedt. The authors examined 164 older adults with hip fractures and found that the primary composite outcome occurred in 35 (21.3%) patients. The total number of potentially inappropriate medications (PIMs) at discharge was considerably lower in the intervention group than in the usual care group (0.8 ± 0.8 for the intervention group vs. 1.1 ± 1.0 for the usual care group; *P* = 0.03). Even so, no notable differences in the primary composite endpoint were found between the intervention and usual care groups (7 in the intervention group and 28 in the usual care group, odds ratio 1.04, 95% CI 0.41–2.65; *P* = 1.00).

Although the intervention to improve appropriate polypharmacy was associated with reducing PIMs, it did not enhance primary and secondary outcomes. Primary outcomes included death and the first occurrence of any subsequent fracture. Secondary outcomes included any new fractures, cardiovascular events, delirium, adverse drug reactions, in-hospital infections, or unplanned hospital admissions. In other words, interventions focusing solely on polypharmacy may not be sufficient for enhancing recovery in older adults with hip fractures. These interventions could be done by a single clinician as long as they are able to review the patient chart and medication history and make clinical decisions to exclude unnecessary medications.

#### Impact of healthcare-multidisciplinary based medication review

Henriksen’s studies attempted to review doctor and clinician attitudes toward medical reconciliation or medication review in hip fracture patients. However, this study provided limited insights for our review, as patient outcomes were unclear. Henriksen found that almost none of the patients had a medication review prior to this study, and a large number of doctors procured lists that were not necessarily in accordance with guidelines. There were reports of only two patients documenting the completion of a medication review in Henriksen’s study.

Arshi’s studies were more general. Arshi used the Standardized Hip Fracture Program (SHFP) as an intervention program, which included medication review, admission checklists, perioperative order sets, multidisciplinary evaluation and management, postoperative rehabilitation goals, and discharge criteria. Patients in the SHFP group had a lower risk-adjusted incidence of postoperative Deep Vein Thrombosis (DVT) within 30 days [0.8% vs. 1.7%, OR 0.48 (0.32–0.72), *P* < 0.001], lower rates of discharge to inpatient facilities instead of home [77.3% vs. 81.5%, OR 0.72 (0.63–0.81), *P* < 0.001], and curbed hospital readmission rates within 30 days [7.1% vs. 9.1%, OR 0.83 (0.71–0.97), *P* = 0.023]. In contrast, SHFP had no major impacts in terms of mortality [6.6% vs. 6.5%, OR 0.97 (0.81–1.18), *P* = 0.777].

Sjoberg and Wallestedt’s studies were relevant due to their design. A medication review was conducted by a geriatrician for each intervention participant, beginning with a systematic assessment of fracture and fall risk. Low-energy fractures were the most common type discovered in the participants (94%). At admission, 26% of intervention and 29% of control participants were treated with fracture-preventing drugs, while 12% and 11%, respectively, were taking bone-active drugs, predominantly bisphosphonates.

After 12 months, 77% of intervention participants and 58% of control participants were taking fracture-preventing drugs (P = 0.01), and 29% and 15%, respectively, were taking bone-active drugs (*P* = 0.04), indicating that the increased use of bone-active drugs in the intervention group one year after hip fracture was a significant outcomes. The mean number of fall-risk-increasing drugs per participant was 3.1 (intervention) and 3.1 (control) at admission. At 12 months, it was 2.9 (intervention) and 3.1 (control).

A 12-month follow-up revealed that 23% of participants had passed away, with higher mortality in the intervention group (27%) compared to the control group (19.2%). The authors concluded that the changes in drug treatment did not contribute to their demise, suggesting that medication reviews did not adversely affect mortality. This implies that external factors, rather than the intervention, influenced outcomes. This finding also suggests that while the intervention contributed positively in certain ways, it may not significantly impact hip fracture outcomes, given that most participants had multiple comorbidities. All these studies focused on teamwork and multidisciplinary approaches within hospitals or healthcare facilities. All these studies were subsequently summarized in Table 1.

**Table 1:** Summary of included studies

References	Population	Intervention	Comparison	Clinical outcomes	Design
Fluck et al. [13]	1105 patients aged above 60 with hip fractures	Identification of polypharmacy, including anticholinergics, and their impact on hip fracture patients	No medication review or intervention	Polypharmacy and ACB in patients with hip fractures are associated with LOS in the hospital One week or longer hospitalization: 55.1% for level 1 (ACB Score = 0 and < 4 drugs a day), 76.6% for level 2 (ACB Score ≥ 1 or ≥ 4 drugs a day), and 80.9% for level 3 (ACB Score ≥ 1 and ≥ 4 drugs a day) Two weeks or longer hospitalization: 19.8% (level 1) to 31.5% (level 2) and 38.1% (level 3) Unable to move within one-day post-surgery rose from 23.2% (level 1) to 36.2% (level 2) and 43.2% (level 3) Comorbidities, such as dementia, stroke, ischemic heart disease, and diabetes), further accentuated by failure to mobilize within 1-day after surgery and pressure ulcers	Retrospective observational study
Henriksen et al. [6]	50 hip fracture patients’ medical records from South-East Norway hospital with a relatively balanced gender distribution of 52% female	Medication management identification of patients with hip fractures	No medication review or intervention	Unclear patients’ outcomes. Still, in the survey, 79% of clinicians reported conducting reconciliation, 37% noted missing medication lists after transitions, and 86% agreed more reviews would benefit patients	A descriptive study using a self-administered clinician survey questionnaire and a retrospective review of hospital records of patients with hip fractures
Arshi et al. [14]	A total of 9360 hip fracture patients were identified from the American College of Surgeons National Surgical Quality Improvement Program, of whom 5070 (54.2%) were treated under a documented SHFP. The median age was 84 years, and 69.9% of patients were women	Standardized Hip Fracture Programs (SHFP), including medication review through a multidisciplinary evaluation	No medication review or intervention	Patients with SHFP had a lower risk-adjusted incidence of postoperative DVT within 30 days [0.8% vs. 1.7%, OR 0.48 (0.32–0.72), * P* < 0.001], discharge to inpatient facilities facility instead of home [77.3% vs. 81.5%, OR 0.72 (0.63–0.81), * P* < 0.001], rates of hospital readmission within 30 days [7.1% vs. 9.1%, OR 0.83 (0.71–0.97), * P* = 0.023]; conversely, SHFP was not significant in terms of mortality [6.6% vs. 6.5%, OR 0.97 (0.81–1.18), * P* = 0.777]	Retrospective cohort study
Komagamine and Hagane [4]	164 patients aged 65 and above with hip fractures	Screening and intervention to reduce polypharmacy in hip fracture patient	No medication review or intervention	The intervention to improve appropriate polypharmacy was associated with a reduction in PIMs but not an improvement in clinical outcomes The total number of potentially inappropriate medications at discharge was markedly lower in the intervention group than in the usual care group (0.8 ± 0.8 for the intervention group vs. 1.1 ± 1.0 for the usual care group; * P* = 0.03) No significant differences in the primary composite outcome were found between the intervention and usual care groups (7 in the intervention group and 28 in the usual care group, OR 1.04, 95% CI 0.41–2.65; * P* = 1.00)	Retrospective observational study
Sjoberg and Wallestedt [15]	199 patients aged 65 and above with hip fractures	A medication review performed by the physician conveyed either orally or written	No medication review or intervention	The study found no significant differences in hard clinical endpoints, including fracture incidence, hospitalizations, or mortality, between the intervention and control groups. While medication use improved, this did not translate into measurable favorable health effects. The mean number of fall-risk-increasing drugs per participant decreased slightly from 3.1 to 2.9 in the intervention group but remained at 3.1 in the control group (*P* = 0.62), indicating no meaningful reduction in medication-related fall risk. Despite these findings, the intervention was well received by physicians, with a median rating of 5 (IQR 4–6) for the oral component and 5 (IQR 4–5.5) for the text component. Nonetheless, the overall results suggest that increased medication adherence alone was not sufficient to significantly impact clinical outcomes such as fractures or hospitalizations	Randomized controlled study
Wagner et al. [16]	93,558 Medicaid enrollees aged 65 years or older. 51,529 in New York and 42,029 in New Jersey	Statewide policy on the review of benzodiazepines used by clinicians	No medication review or intervention	The triplicate prescription policy has successfully led to a major reduction in benzodiazepine prescriptions but did not have a notable impact on the risk of hip fractures	Quasi-experimental

*PIMs* potentially inappropriate medications, *ACB* Anticholinergic Agents Burden, *LOS* Length of Stay

#### Impact of policy and state-based level of medication review

Wagner’s studies provide significant insights into a statewide policy in the United States that aimed to encourage clinicians to reassess the use of benzodiazepines. This approach addresses the issue of benzodiazepine misuse and its potential adverse effects, such as hip fractures, particularly in older adults. The authors highlighted that the policy successfully led to a substantial reduction in benzodiazepine prescriptions, marking a positive step toward improving patient safety.

**Fig. 3:**
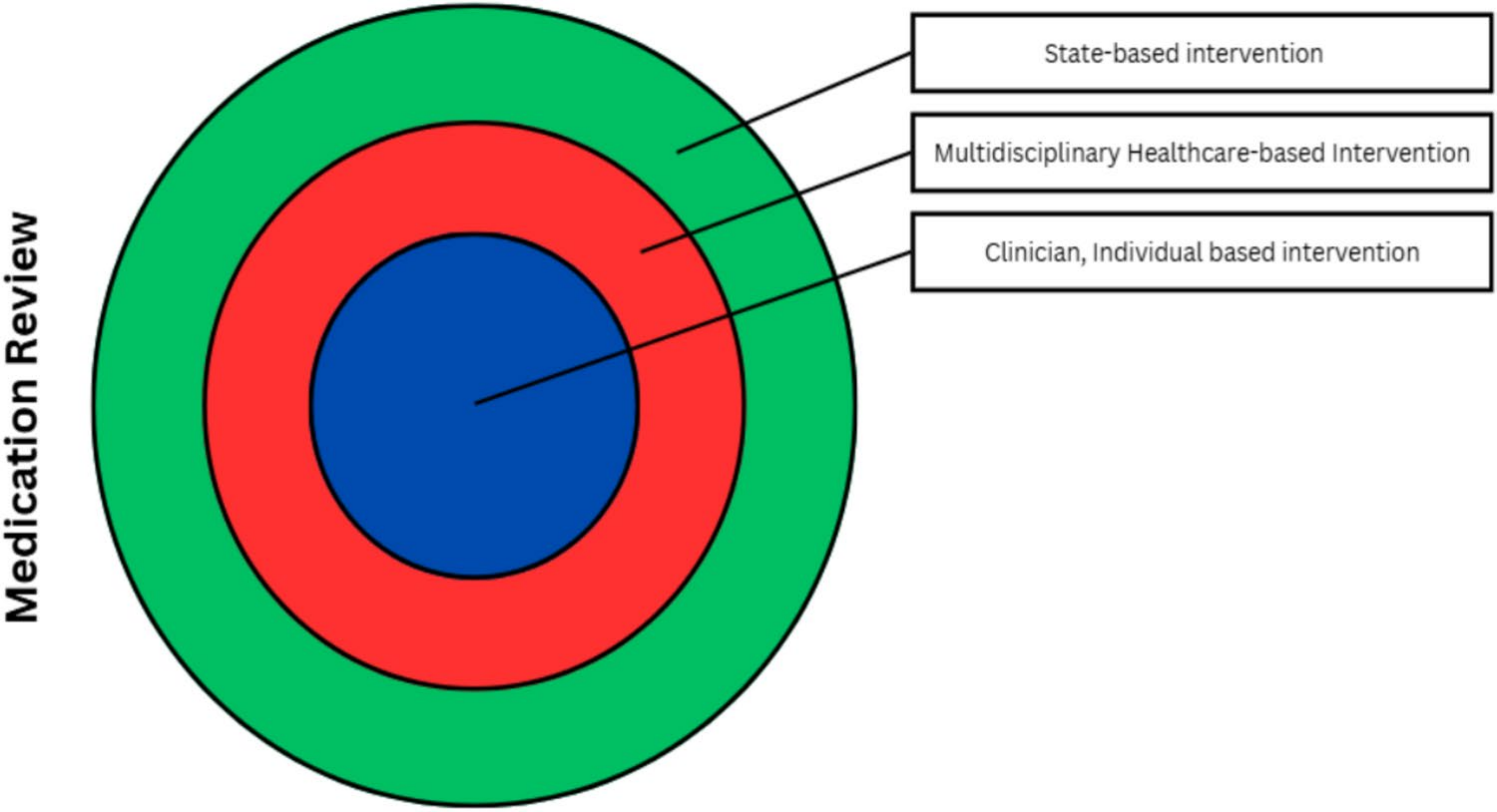
Venn-diagram adaptation of medication review approach to create an impactful intervention

However, hip fracture rates remained stable, suggesting that while the policy is making progress and opens the door for further exploration of other factors contributing to hip fractures among older adults, it also highlights the limitations of policy-driven interventions that target a single risk factor without addressing broader underlying causes of hip fractures. All these elaborations were further summarized in Fig. 3.

## Discussion

Older adults constitute the largest consumers of prescription medications worldwide [17, 18]. Age-related physiologic changes, such as impaired hepatic and renal function and increased total body fat, heighten their vulnerability to adverse drug reactions. These changes make prescribing and monitoring pharmacologic therapies in this demographic particularly complex. The prevalence of drug–drug and drug–disease interactions in older adults further exacerbates risks, leading to falls, hospitalizations, and diminished quality of life [19–21]. Polypharmacy is common among older individuals, often resulting in inappropriate prescribing, worsening drug-related adverse effects, and increased healthcare costs [22, 23]. Therefore, medication reviews are essential to prevent medication-related complications [24, 25].

Medication reviews can be conducted either during acute hospital stays or periodically in outpatient settings to reassess the appropriateness of long-term pharmacotherapy [26]. According to established definitions, a medication review is a structured process employing explicit (criteria-based), implicit (judgment-based), or hybrid tools to identify PIPs. Explicit prescribing criteria help detect inappropriate medications, prescribing omissions, and mis-prescriptions. Several established compendiums for this purpose involve the Beers Criteria, the FORTA list, the STOPP-J, the PRISCUS list, the NORGEP criteria, and the Laroche list [27–32]. These frameworks have been widely utilized in many geriatric settings to enhance medication safety. Even so, only a few studies have explored their impact on hip fracture patients, necessitating a dedicated systematic review.

To our knowledge, this is the first systematic review to assess the impact of medication reviews on clinical outcomes in older adults with hip fractures. The absence of previous reviews emphasizes the novelty and relevance of our study. Given the increasing recognition of medication optimization as a crucial component of postoperative care in geriatric populations, our findings address an important gap in the literature. Previous systematic reviews on medication reviews in older adults have mainly focused on general geriatric populations rather than specific cohorts such as hip fracture patients. For instance, a meta-analysis by Rankin et al. (2018) demonstrated that medication reviews minimized inappropriate prescribing and adverse drug reactions in older adults. That said, no significant impact was observed on hospitalizations or mortality. Our study aligns with these findings, indicating that while medication review interventions optimize pharmacotherapy, their direct effect on mortality remains inconclusive. Future studies with rigorous methodologies are warranted to confirm and expand upon these results.

A critical aspect of our study involved assessing the RoB across included trials, as methodological quality variations may influence the reliability of pooled evidence. Our RoB evaluation indicated concerns primarily in selection and performance bias. Notably, Arshi et al. [14] exhibited an unclear or high risk in random sequence generation and allocation concealment, raising concerns about potential bias in treatment assignment. Likewise, inadequate blinding of participants and personnel in multiple studies introduced performance bias that may have influenced the evaluation of findings. In contrast, detection and attrition biases were generally low, suggesting that follow-up completeness and outcome assessments were adequately managed. The potential for selective reporting bias remains a concern, particularly due to some studies’ absence of pre-registered protocols. These methodological limitations should be acknowledged when interpreting our findings, as they may contribute to heterogeneity across studies. Future research should strictly adhere to randomized controlled trial guidelines to enhance evidence robustness.

Medication reviews have been extensively evaluated in older adults with multimorbidity and polypharmacy, with endpoints focusing on drug-related hospitalizations, inappropriate medication use, clinically significant drug–drug interactions, health-related quality of life, length of hospital stay, and pain relief. Several studies have demonstrated that medication reviews can reduce inpatient prescriptions and improve medication appropriateness [26, 33].

Polypharmacy was more prevalent in older adult hip fracture patients (74.8%) compared to the general older population. These findings highlight the necessity of evaluating treatment regimens and medication burden. Furthermore, Fluck’s study also showed that patients with higher medication status levels had prolonged hospital stays, increased difficulty mobilizing postoperatively, and a heightened risk of pressure ulcers [13]. These findings align with our review, emphasizing optimizing medication regimens in this vulnerable group.

Several studies yielded consistent findings regarding the benefits of medication reviews. The intervention group showed significant improvement following comprehensive medication evaluations [34, 35]. This progress is reflected in a notable decrease in PIMs and a higher percentage of patients receiving osteoporosis treatment upon hospital discharge. The reduction of PIMs is particularly beneficial, as medications in hip fracture patients are associated with an increased risk of falls and fractures [35], elevated mortality rates [36], higher rates of rehospitalization, and adverse drug reactions [34, 35].

Contrary to these findings, Komagamine’s study reported no significant reduction in composite outcomes following a polypharmacy-targeted intervention, with an odds ratio of 1.04 even after adjusting for baseline confounders [4]. Furthermore, Sjoberg’s study also found no statistically meaningful differences between intervention and control groups regarding falls, fractures, or mortality at a 12-month follow-up. Although medication reviews curtailed medication-related problems and dose adjustments, they did not translate into improved survival or quality of life [15]. Several factors may explain these results. First, while comprehensive geriatric assessments and acute geriatric care units have yielded clinical advantages in hospitalized older adults, an intervention solely targeting polypharmacy may lack efficacy in improving broader patient outcomes. Second, the failure to achieve meaningful clinical improvements may stem from incomplete adjustments in inappropriate medication use [4].

These results are consistent with our findings, as we observed no marked correlation between medication reviews and mortality in older adults with multiple comorbidities. While medication review interventions did not intensely result in the reduction of mortality rates, they did demonstrate benefits in mitigating postoperative complications and shortening hospital stays among the geriatric population [13, 18].

Given the high prevalence of polypharmacy among hip fracture patients, it is essential to conduct systematic reviews on medication review strategies. Optimizing and minimizing pharmacotherapy could significantly enhance recovery and rehabilitation outcomes in post-fracture care. There is a notable absence of systematic reviews specifically addressing medication reviews for older adults with hip fractures, highlighting a significant gap in the existing literature. Our study highlights the significance of structured medication management in health improvements and calls for further research employing rigorous methodologies to assess the long-term clinical and economic impacts of medication reviews in this high-risk population. A comprehensive understanding of medication optimization can ultimately facilitate better healthcare resource allocation and enhance patient care quality in geriatric settings.

## Conclusion

In conclusion, conducting medication reviews shows positive outcomes, including a reduction in potentially inappropriate medications (PIMs), which leads to lower adverse outcomes in older adults with hip fractures, such as shorter hospital stays, and lower incidences of postoperative complications, including DVT, stroke, and ischemic heart disease. However, these interventions did not result in a significant mitigation of mortality rates among this vulnerable population.

## Limitation

The limitations of our studies are the number of included studies and the heterogeneous endpoints of the respective studies. These limitations mean that sufficient and proper meta-analysis could not be performed. While our study was able to conclude a recommendation, it may be imperative to perform further studies to legitimize the result.

## Data Availability

All data supporting the findings of this study are available within the paper.
